# Surgical Treatment for Posterior Dislocation of Hip Combined with Acetabular Fractures Using Preoperative Virtual Simulation and Three-Dimensional Printing Model-Assisted Precontoured Plate Fixation Techniques

**DOI:** 10.1155/2019/3971571

**Published:** 2019-02-28

**Authors:** Yuan-Ta Li, Chun-Chi Hung, Yu-Ching Chou, Jia-En Chen, Chia-Chun Wu, Hsain-Chung Shen, Tsu-Te Yeh

**Affiliations:** ^1^Department of Orthopaedic Surgery, Tri-Service General Hospital and National Defense Medical Center, Taipei, Taiwan; ^2^School of Public Health, National Defense Medical Center, Taipei, Taiwan; ^3^Department of Biomedical Engineering, National Defense Medical Center, Taipei, Taiwan

## Abstract

**Background and Purpose:**

Hip dislocation combined with acetabular fracture remains a challenging condition for orthopedic surgeons. In this study, we utilized a computer-assisted simulation and three-dimensional (3D) printing technology to treat patients with hip dislocation combined with acetabular fracture. We hypothesized that the 3D printing-assisted method would shorten the internal fixation time and surgical time.

**Methods:**

We retrospectively reviewed 16 patients diagnosed with traumatic posterior dislocation of hip combined with acetabular fractures and treated with plate fixation from September 2013 to August 2017. Patients were divided into two groups: (1) traditional method and (2) 3D printing groups. In the traditional method group, the plates were contoured during the surgery, whereas in the 3D printing group, the patient's pelvic computed tomography image was transformed to the 3D medical image software for processing preoperatively. The fracture reduction was simulated by the computer. Thereafter, the 1:1 scale 3D printing model was used to design the surgical plan and contour patient-specific plates preoperatively.

**Results:**

The internal fixation time was significantly shorter in the 3D printing group than in the traditional method group (-33 min,* P*<0.05). The mean operative time was shorter than that in the traditional method group (-43 min). However, blood loss and postoperative radiograph results were similar between the groups. The complication rate was lower in the 3D printing group (2/7) than in the traditional method group (5/9).

**Interpretation:**

Computer-assisted simulation with 3D printing technology is a more efficient method for treating hip dislocation combined with acetabular fractures.

## 1. Introduction

Hip dislocations combined with acetabular fractures are typically caused by high-energy trauma, usually from motor vehicle accidents. Fractures of the posterior wall of the acetabulum are one of the most common types of acetabular fractures, accounting for up to 30% of all acetabular fractures [1]. These severe injuries often require urgent closed reduction of the hip and surgery to restore hip joint stability and articular surface anatomically. Anatomic reduction of acetabulum is an important factor to prevent posttraumatic osteoarthritis, thereby improving the patients' long-term functional outcome [2]. However, fractures of the acetabulum continue to be a challenge for orthopedic surgeons. The surgery is complex and demanding even for the experienced surgeon [3]. Stable and anatomic fixation for fractured posterior wall and column can provide hip stability. Given the morphological variations among individuals and varied acetabular fracture patterns, creating a universal and anatomical contoured fixation plate suitable for every patient is difficult.

The possible medical applications of preoperative virtual simulation and three-dimensional (3D) printing have grown, as this technology has become more accessible financially and technically. The use of a computerized virtual planning system in acetabular fracture surgery is a valuable tool for the surgeon to better understand the fracture pattern [4]. Printed 3D models have been used for preoperative planning and manufacturing of surgical guides and joint implants. Upex et al. [5] reported a surgical technique for treating fractures of both columns of the acetabulum with precontouring plates obtained using the 3D-printing model. Hence, in this study, patients with acetabular fractures combined with hip dislocation were treated using traditional plate fixation methods or using preoperative contoured plates in the 3D printing model. We hypothesized that preoperative contoured plates can reduce operation and instrumentation times during the surgery. We also aimed to compare surgical and clinical outcomes between the traditional and 3D printing simulation methods.

## 2. Materials and Methods

### 2.1. Patients

**Figure 1:**
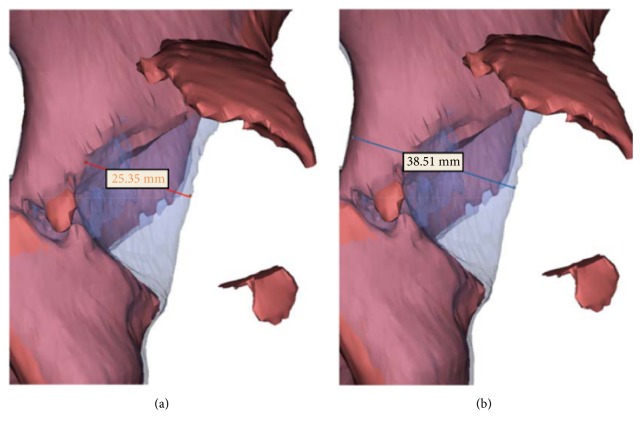
We mirror the healthy hemipelvis to be the substrate and overlap the fractured portion using the transparent method. Therefore, the size can be estimated from the posterior view by measuring the maximal length from the edge of the posterior wall to the fractured edge (a) divided by the length from the edge of the posterior wall to the border of the posterior column (b). Therefore, the estimated percentage of posterior wall fragment is 25.35/38.51=65.82%.

We retrospectively included 16 patients who were diagnosed with traumatic dislocation of the hip joint combined with acetabular fractures from September 2013 to August 2017. The institutional review board at our hospital approved this study. All patients signed an informed consent form before surgery. They were all treated at our institution by a single experienced surgeon. The inclusion criterion was undergoing open reduction for acetabular fracture and internal fixation with plates. The exclusion criteria were (1) stable hip joint by examination under anesthesia after closed reduction of the hip, (2) acetabular fractures fixed with other implants, (3) fracture dislocations with concomitant preoperative neurovascular injury, and (4) acetabular fractures combined with pelvic iliac wing fractures. These 16 patients were divided into two groups: (1) traditional method (patients treated from September 2013 to October 2016) and (2) 3D printing (patients treated from November 2016 to August 2017) groups. The acetabular fracture pattern was categorized according to the Letournel-Judet classification [6]. Moreover, posterior dislocation of the hip was categorized according to the Thompson and Epstein classification. The percentage of posterior wall fragment size was evaluated through a 3D-reconstructed model using a 3D medical image processing software (Materialise Mimics, version 19, Belgium) (Figure 1). Patient characteristics are presented in Table 1.

### 2.2. Surgical Technique

All patients underwent closed reduction of hip joint in the operation room on the day of injury. The examination under anesthesia was performed to evaluate the stability of the affected hip joint. Distal femoral skeletal tractions were all applied because of instability. All patients underwent pelvic computed tomography (CT) using 3-mm slices. We used the Kocher-Langenbeck approach for all patients to treat acetabular posterior wall or posterior column fractures.

In the traditional method group, the plates were contoured during the operation. After reduction of the fractured site, a plastic aluminum plate as a template was adjusted by pressing it along the curvature of the acetabulum. One-third of the tubular plate was bent as a spring plate, followed by overlapping of the reconstruction plates as posterior wall buttress plates or posterior column plates according to the shape of templates.

**Figure 2:**
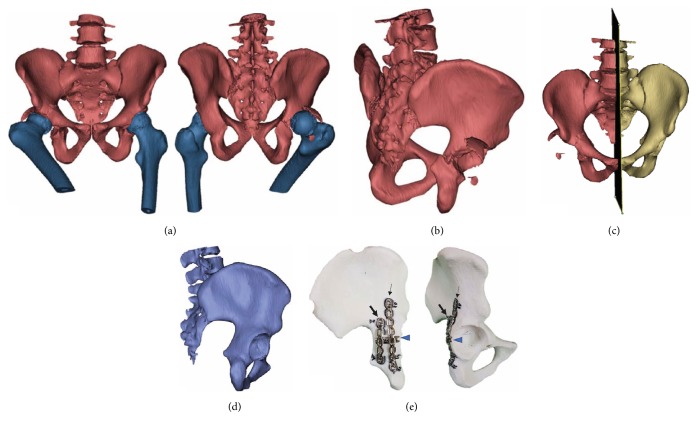
Computer-assisted simulation with three-dimensional (3D) model for designing plate fixation: (a) 3D image reconstruction by Mimics with segmentation (anteroposterior and posteroanterior views). (b) Image with femur subtraction. (c) Setting the midline as mirrored plane for virtual reduction. (d) Mirrored nonfractured hemipelvis. (e) A 3D model of the mirrored nonfractured hemipelvis and design of the H-shaped configuration internal fixation. One spring plate (arrowhead) covered by posterior wall buttress plate (arrow) and posterior column (bold arrow).

In the 3D printing group, we set up a preoperative planning protocol (Figure 2). First, we input the patient's pelvic CT image (DICOM format) into the 3D medical image processing software (Materialise Mimics). According to the variant threshold, we can separate the bony part from the soft tissues. With the function of separation, the femoral head and proximal femur can be erased to show the joint surface of the acetabulum. Thereafter, the patient-specific 3D image was reconstructed. For simulating the anatomic reduction of the fractured acetabulum, mirroring the contralateral noninjured hemipelvis as the repositioned model is a feasible method. At the same time, the percentage of defect of the posterior wall can be estimated using a rematch method. The simulation process was performed by a single orthopedic surgeon who is qualified to perform 3D printing engineering. Finally, the virtual hemipelvis model was exported in a stereolithography format for 3D printing fabricated by a fused deposition modeling desktop machinery (UP BOX+, Tiertime, China, or Mass Portal XD 40, Mass Portal, Latvia). We used the 3D printing model to design the surgical plan (including the type of plate and plate number, curvature, position, and screw length). The precontoured plates were sterilized preoperatively and were applied immediately after an adequate reduction.

In patients with combined femoral head fractures, a surgical intervention (open reduction and internal fixation with headless screws) was performed using the Smith-Petersen approach in the other stage of surgery, if indicated. In patients with anterior column involvement (transverse or T-shape type), staged internal fixation with plate surgery was performed using the anterior approach to enhance the stability of the acetabulum, if indicated.

The rehabilitation program, including isometric quadriceps strengthening and hip passive motion, was started 1 week after the surgery. Nonweight-bearing training was prescribed about 4 weeks postoperatively. Partial weight-bearing training with crutches was permitted when radiography results during the outpatient follow-up indicated a partial callus formation. Full weight-bearing was tolerated at 2 months postoperatively.

### 2.3. Radiological Outcome and Complications Evaluation

**Figure 3:**
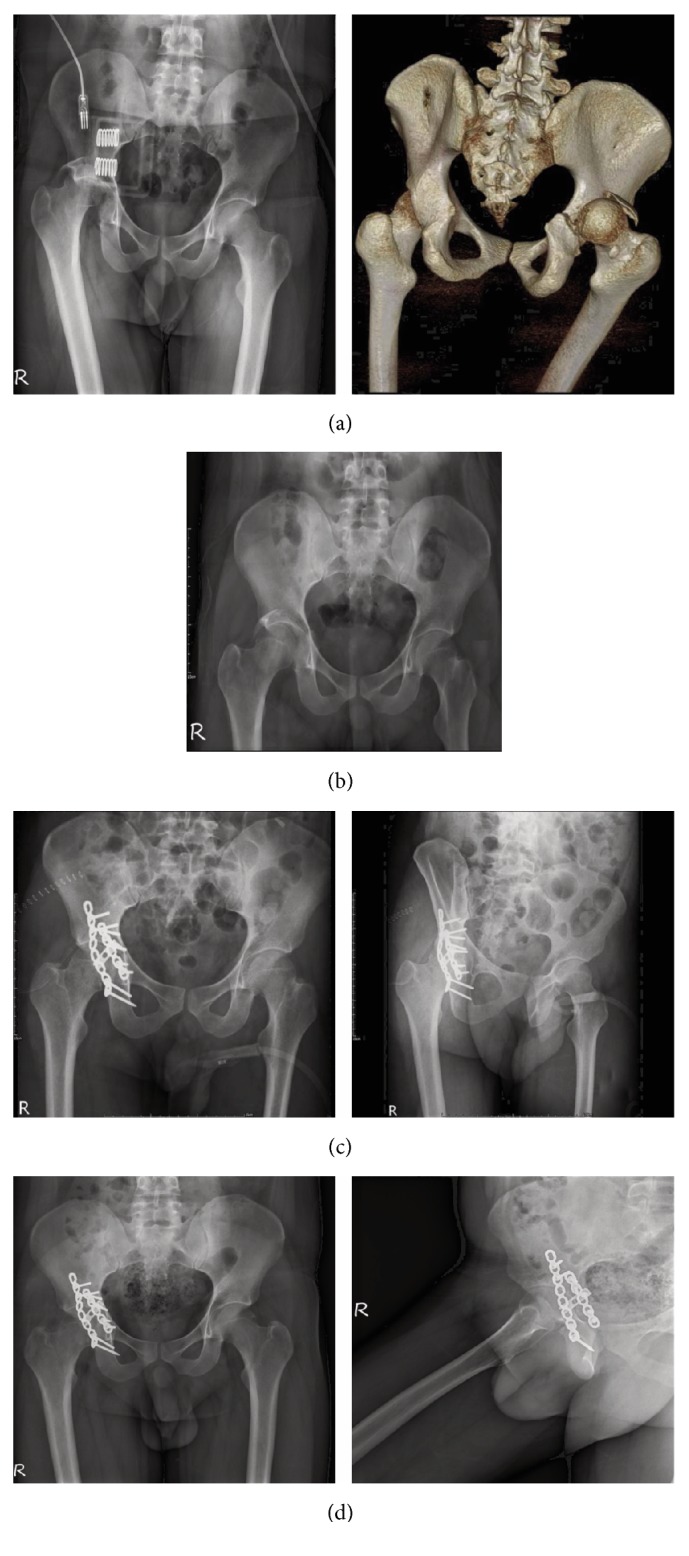
Images of posterior hip dislocation combined with transverse-type and posterior wall fracture of the right acetabulum in a 23-year-old man. (a) The posteriorly dislocated hip with acetabular fracture is shown by X-ray and 3D computed tomography images. (b) Status after closed reduction of the right hip with application of skeletal traction. (c) Open reduction via the Kocher-Langenbeck approach and internal fixation with three precontoured plates (anteroposterior and Judet views). (d) Postoperative follow-up radiographs at 4 months (hip anteroposterior and lateral views).

**Figure 4:**
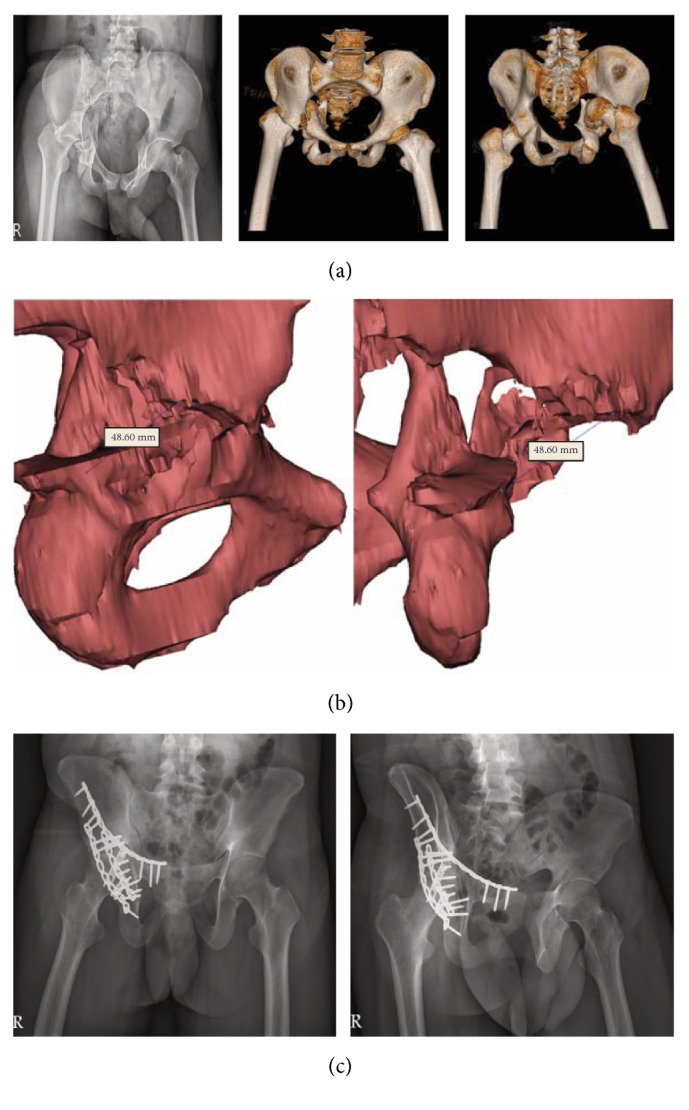
Images of posterior hip dislocation combined with acetabular fracture (T-shape + posterior wall) of the right acetabulum in a 22-year-old man: (a) preoperative pelvic plain film and CT scan film. (b) The posterior wall fragment length is about 48.60 mm as measured by the software. (c) The postoperative pelvic plain film reveals two spring plates, one posterior wall buttress plate, one posterior column plate, and one anterior column plate fixation.

In the 3D printing group, we analyzed the preoperative course, including software processing, 3D model printing, and plate precontouring times. Intraoperative parameters, including operation time, instrumentation time, and blood loss, were compared between the two groups. Postoperative X-ray film [anteroposterior (A-P) and Judet views] was used to evaluate the quality of reduction. All follow-up radiographs were evaluated by three orthopedic surgeons, and the discrimination of fracture reduction was determined by a consensus. The quality of fracture reduction of the acetabulum was graded as good (0-2-mm displacement) or fair (≥ 2-mm displacement). Complications included surgical wound infection, neurovascular injury during surgery, implant loosening, screw penetration into the hip joint, loss of reduction, posttraumatic arthritis, and avascular necrosis (AVN) of the femoral head. Two cases in the 3D printing group are shown in Figures 3 and 4.

### 2.4. Statistics

Quantitative data were expressed as means ± standard deviation. Statistical analyses were performed using the Statistical Package for the Social Sciences software (version 22, SPSS, Inc., Chicago, IL, USA). The independent *t* test or chi-squared test was used for comparison of operation time, internal fixation time, and blood loss. Fisher's exact test was used for assessment of radiological results. A *P* < 0.05 was considered statistically significant.

## 3. Results 

### 3.1. Clinical Data

Patients' characteristics are summarized in Table 2. The demographics were homogeneous between the two groups (all *P* > 0.05). The mean follow-up duration was 19.00 ± 13.31 and 9.29 ± 4.86 months in the traditional method and 3D printing groups, respectively (*P* = 0.02).

One and two patients underwent open reduction and internal fixation for femoral head fracture in the traditional method and 3D printing groups, respectively. In the 3D printing group, anterior column fixation with plate surgery was performed in two cases.

### 3.2. Perioperative Clinical Parameters

Preoperative and intraoperative parameters are shown in Table 3. In the 3D printing group, the mean software simulation time was 11.14 ± 1.07 min. The mean time of 3D printing of the 1:1 scale hemipelvic model for precontouring was 608.43 ± 27.54 min. The mean plate precontouring time was 46.86 ± 17.69 min. The mean operation time of the 3D printing group was shorter (43 min) than that of the traditional method group. There was no significant difference in blood loss during surgery between the two groups. However, the instrumentation time was significantly longer in the traditional method group than in the 3D printing group (71.43 vs 38.43 min, *P* < 0.001).

### 3.3. Postoperative Radiological Evaluation

Two patients (22.2%) had over 2-mm displacement of the articular surface in the traditional method group. In the 3D printing group, the displacements were all < 2 mm. The postoperative radiological results between the two groups were similar (*P* = 0.475).

### 3.4. Complications

In the traditional method group, three patients had posttraumatic arthritis, one patient sustained AVN of the femoral head 9 months after injury, and one patient had heterotopic ossification within 1 month after the operation. In the 3D printing group, two patients suffered complications. One patient sustained a superior gluteal artery injury intraoperatively (blood loss of 1900 ml) and AVN of the femoral head (18 months after injury), whereas the other patient had AVN of the femoral head (3 months after injury). Surgical wound infection, implant loosening, screw malposition, or loss of reduction was not observed in both groups.

## 4. Discussion

Hip fracture-dislocation is a high-energy trauma with a low incidence rate. Ahmed et al. reported that posterior wall facture associated with posterior hip dislocation occurred in about 5.4% of cases [7]. Given the complex anatomy of the acetabulum and limited information from the plain radiography, the acetabular fracture is the most challenging fracture to manage. 3D-printing technology plays an important role. Awan et al. [8] utilized 3D-printed models of complicated acetabular fractures to attain short-term understanding of fracture patterns and clarify the classification system. Moreover, Kim et al. [9] emphasized that the 3D-printing patient-specific model provides surgeon of the detailed anatomical information, preoperative planning, and education for surgical trainees. Some specialists can make customized osteosynthesis and surgical jigs via the 3D-printing technique. Maini et al. [10] designed an anatomical posterior column plate for Indian origin via virtual simulation and 3D-printed plastic template to match the surface, and good quality of reduction was reported with this method. Merema et al. [11] claimed that 3D printing and patient-based surgical guides are achievable and auspicious for the operative treatment of acetabular fractures. Hsu et al. [12] reported the efficacy of virtual simulation and 3D-printing method before acetabular surgery, which can reduce the surgical duration, instrumentation time, and blood loss. Hence, in this study, we utilized a computer-assisted simulation and 3D printing technology to treat patients with hip dislocation combined with acetabular fracture. We observed that the 3D printing-assisted method shortened the internal fixation and surgical times. Thereupon, computer-assisted simulation with 3D printing technology is a more efficient method for treating hip dislocation combined with acetabular fractures. Computer-assisted simulation using CT scan data could help surgeons in preoperative planning [13]. 3D CT images can provide a precise fracture pattern and increase the accuracy of acetabular fracture classification [14]. However, the image of the fractured acetabulum is sometimes covered by the femoral head, especially when the hip is dislocated. Using the software, the femoral head can be eliminated, and complete evaluation of the acetabular fracture pattern can be achieved [15–17]. Posterior wall fragment size is one of the risk factors for the residual instability of hip joint after closed reduction [17]. However, there was no uniform method to calculate the percentage of posterior wall involvement. Several studies had described measurement methods by using a static two-dimensional CT image [18, 19]. In this study, we proposed a new method by using simulation of the contralateral uninjured acetabulum to assess the size of the fracture fragment in a 3D structure. This method allowed evaluating the posterior column plate fixation according to the simulation result. In our cases, the posterior column plate was used in the following indications: (1) fractures with posterior column involvement and (2) posterior wall fragment size > 45%. The use of posterior column plate to buttress the large posterior wall fracture is more stable, and no reduction losses were observed in this study.

Acetabular fracture pattern evaluation and preoperative planning can be achieved by using a 3D printing acetabular model. 3D printing modeling of real acetabular fractures can assist surgeons in understanding the characteristics of complex fractures prior to surgery to significantly reduce the degree of interobserver variability in fracture classification [20]. In this study, we printed the patient-specific models of acetabular fractures in some complicated cases and fracture-reduced acetabular models in all cases. All processes were performed by a single orthopedic doctor within 24 h. The software time in our study was around 11 min, and the 3D printing time for the hemipelvis was about 10 h. The manufacturing time for the 3D model was efficient in this study.

For achieving good functional and radiographic outcomes, an adequate and stable internal fixation to maintain anatomic reduction is a key [2]. Li et al. [21] reported that the internal fixation of two parallel reconstruction plates, which facilitated a rigid fixation and avoided fracture fragment injury, was an effective and reliable method in treating fractures of the posterior wall of the acetabulum. For fixing comminuted posterior wall fractures combined with marginal fragments, customized spring plates can be a suitable method of adjunctive fixation [22]. Liu et al. [23] also reported that combined plate internal fixation for posterior wall fractures of the acetabulum was stable and reliable. According to the reports of Richter et al. [24] and Lee et al. [22], the spring plate acts as a dynamic buttress for periarticular fragments biomechanically. It can provide solid fixation for posterior wall fracture of the acetabulum, especially in comminution or small fragment cases. A spring plate overlapped by a posterior buttress plate is also known to improve fixation strength. We contoured the spring plate using one-third tubular plate, posterior wall buttress plate, and posterior column plates with 3.5-mm reconstruction locking plates preoperatively on the patient-specific acetabular models. The number and position of the plates were designed according to the 3D model. The plates were fixed on the model, and the length of the screws can be estimated before the surgery. In this study, the number of spring plates was dependent on the length (in a cephalad to caudal direction) of the posterior wall fragment. If the length of the fragment is over 40 mm measured using the software, two spring plates were used to cover the fragment. Then, a posterior wall buttress plate or posterior column plate was used to overlap the previous spring plates to obtain adequate coverage and stability. The 3D-printing technology, including 3D image reconstruction, can also offer great advantage in evaluating the fracture pattern of complicated acetabular injury. For comminution cases, the 1: 1 model reflected the severity of injury, which can be outputted via 3D printer to promote realization of the anatomical relationship. For impaction cases, we can restore the articular surface virtually and evaluate the amount of bone grafting before operation.

In the traditional method, contouring the posterior wall plate was time consuming and required more extensive soft tissue dissection. In this study, the instrumentation time was significantly shorter in the 3D printing group than in the traditional method group because of the utilization of precontoured plates, which can decrease recontoured times during operation. According to the reports by Maini L et al. [25, 26], patient-specific precontoured plate made using rapid prototyping model is not only a better implant than intraoperatively contoured plate, but can also improve the outcomes of acetabular fracture surgery. Hung et al. [27] compared the surgical and instrumentation times to treat anterior pelvic ring fractures between the conventional and 3D printing groups. The instrumentation time was significantly shorter in the 3D printing group. They also reported that minimally invasive incision could be achieved when using precontoured plate in treating anterior pelvic fracture, and a decreasing trend in surgical time was noted in the 3D group. In our study, the mean operation time of the 3D printing group was shorter than that of the conventional group, but without significant difference, which may be due to the same surgical approach method used in both groups or the small sample size in the 3D printing group. More cases should be collected in future studies.

The blood loss between two groups was similar. Injury of the superior gluteal artery was noted in one patient in the 3D printing group. Excluding this case, the average blood loss was lesser in the 3D printing group than in the traditional method group. Besides, one case in the 3D printing group developed AVN of the femoral head within 3 months after the surgery. However, these events were not associated with the application of the 3D printing method.

The early reduction of a dislocated hip and quality of surgical reduction were strong positive predictors of functional and radiographic outcomes at follow-up [2]. We analyzed the quality of reduction via a postoperative X-ray including standard A-P and Judet views. There was no statistically significant difference in the radiographic outcomes between both groups. However, good reduction rates were higher in the 3D printing group than in the traditional method group (7 [100%] and 5 [77.8%] patients, respectively), suggesting that the 3D printing method better facilitates fracture reduction in acetabular surgery. The primary complication after acetabular fractures is posttraumatic arthritis. The quality of the fracture reduction appears to be the main determinant of the risk of late traumatic arthritis. Other complications include sciatic nerve injury, heterotopic ossification, and osteonecrosis of the femoral head [28]. In this study, two patients in the traditional method group who had radiologic displacement > 2 mm had posttraumatic arthritis of the affected hip within 18 months, and conversion to total hip replacement was performed. The overall incidence of late traumatic arthritis and AVN of the femoral head was 19% (3/16) in this study.

In this study, some patients underwent another stage surgery for femoral head fracture fixation or anterior column fracture fixation in combined transverse or T-type injury. Flip osteotomy of the greater trochanter with surgical dislocation of the hip can achieve complete reduction of the fractured femoral head and application of internal fixation in the same stage. However, we do not want to introduce iatrogenic fracture and apply additional internal fixation in the greater trochanter. With the Smith-Petersen approach, the femoral head can be exposed directly with Figure 4 position (abduction and external rotation) of the affected hip, without dislocation of the femoral head. Anterior plating fixation in transverse or T-type fracture was performed based on Becker's biomechanical study [29]. They compared minimally invasive screw fixation and anterior plating in the treatment of acetabular T-type fractures. The anterior locking plate provided more stability than column screw fixation, and the least displacement was observed in the plate group. We decided to perform 3D printing model-assisted precontoured locking plate fixation in the anterior column of the acetabulum in another stage in our study, and it can be done via mini-invasive approach [27].

This study had some limitations. First, this was a nonrandomized study. Second, it had a relatively small sample size. Third, the study assessed long-term functional outcomes. A larger patient population using this method with a long-term follow-up is needed to further assess the clinical advantages of the 3D printing method.

## 5. Conclusions

The combination of computer-assisted simulation and 3D printing technology can provide an effective method for treating acetabular fractures using precontoured plates, which can provide patient-specific internal fixations to achieve a more anatomic reduction and reduce internal fixation time. Future studies evaluating the quality of reduction of the acetabulum articular surface via a follow-up CT scan and documentation of its long-term functional outcome are warranted.

## Figures and Tables

**Table 1:** Patient profiles.

Patient No.	Group	Age (yr)/Sex	Type of fracture	Letournel classification	Thompson classification	FH fracture	PwSP No.	PwBP No.	PcP No.	AcP No.	Size of Pw (%)
1	1	24/M	Posterior wall	Elementary	III	N	1	1	1	0	84.73
2	1	53/F	Posterior column + posterior wall	Associated	III + IV	N	1	1	1	0	45.91
3	1	36/F	T-shape + posterior wall	N/A	II + IV	N	1	1	1	0	59.26
4	1	22/M	Posterior wall	Elementary	II + V	Y	1	1	0	0	37.42
5	1	56/M	Posterior wall	Elementary	III	N	1	1	1	0	54.35
6	1	32/M	T-shape + posterior wall	N/A	III + IV	N	0	1	1	0	94.29
7	1	24/M	Posterior wall	Elementary	III + V	Y	2	1	1	0	67.33
8	1	66/M	Posterior wall	Elementary	III + V	Y	1	1	1	0	80.06
9	1	20/F	Posterior wall	Elementary	II + V	Y	2	1	0	0	31.62
10	2	22/M	T-shape + posterior wall	N/A	III + IV	N	2	1	1	1	79.34
11	2	31/M	Posterior wall	Elementary	III + V	Y	1	1	0	0	35.49
12	2	23/M	Transverse + posterior wall	Associated	II + IV	N	1	1	1	0	65.82
13	2	37/M	Transverse + posterior wall	Associated	III + IV	N	1	1	1	1	62.78
14	2	26/M	Posterior wall	Elementary	III + V	Y	1	1	0	0	44.79
15	2	63/M	Posterior wall	Elementary	III	N	2	1	1	0	85.46
16	2	23/M	Posterior column + posterior wall	Associated	III + IV	N	0	1	1	0	48.78

AcP, anterior column plate; FH, femoral head; N/A, not applicable; PcP, posterior column plate; Pw, posterior wall; PwBP, posterior wall buttress plate; PwSP, posterior wall spring plate.

**Table 2:** Demographic data.

	Traditional method	3D printing method	P value
	Group 1 (n=9)	Group 2 (n=7)	
Age (year), M±SD	37.00±17.09	32.14±14.63	0.559^a^
Sex, n (%)			0.213^b^
Male	6 (66.7)	7 (100)	
Female	3 (33.3)	0 (0)	
BMI (kg/m^2^)	27.22±2.95	26.29±2.29	0.500^a^
Fracture classification, n (%)			0.515 ^b^
T-shaped + Pw	2 (22.2)	1 (14.3)	
Pc + Pw	1 (11.2)	1 (14.3)	
Pw	6 (66.7)	3 (42.9)	
Transverse + Pw	0 (0)	2 (28.6)	
Affected side, n (%)			1.000^b^
Right	6 (66.7)	5 (71.4)	
Left	3 (33.3)	2 (28.6)	
Pw fragment size (%)	61.66±21.69	60.35±18.35	0.900^a^
Dislocation, n (%)	9 (100)	7 (100)	n/a
Femoral head fracture, n (%)			0.633^b^
no	5 (55.6)	5 (71.4)	
yes	4 (44.4)	2 (28.6)	
Number of PwSP, n (%)			1.000^b^
0	1 (11.1)	1 (14.3)	
1	6 (66.7)	4 (57.1)	
2	2 (22.2)	2 (28.6)	
Number of PwBP, n (%)	9 (100)	7 (100)	n/a
Number of PcP, n (%)			1.000^b^
0	2 (22.2)	2 (28.6)	
1	7 (77.8)	5 (71.4)	

Ac, anterior column; AcP, anterior column plate; BMI, body mass index; M±SD, mean ± standard deviation; n, patient number; n/a, not applicable; Pc, posterior column; PcP, posterior column plate; Pw, posterior wall; PwBP, posterior wall buttress plate; PwSP, posterior wall spring plate.

^a^ Independent *t* test or chi-square test.

^b^ Fisher's exact test.

**Table 3:** Clinical characteristics and outcomes.

	Traditional method	3D printing method	P value
	Group 1 (n=9)	Group 2 (n=7)	
Software simulation time (min), M±SD	-* *-	11.14±1.07	n/a
3D printing time (min), M±SD	-* *-	608.43±27.54	n/a
Plate pre-contouring time (min), M±SD	-* *-	46.86±17.69	n/a
Operation time (min), M±SD	254.44±34.46	211.71±52.23	0.069^a^
Instrumentation time (min), M±SD	71.78±9.69	38.43±10.81	<0.001^a^
Blood loss (ml), M±SD	742.22±228.68	735.71±614.22	0.977^a^
Postoperative X-ray film, n (%)			0.475^b^
< 2 mm displacement	7 (77.8)	7 (100)	
> 2 mm displacement	2 (22.2)	0 (0)	
Complication, n (%)			
No	4 (44.4)	5 (71.4)	
Yes	5 (55.6)	2 (28.6)*∗*	

AVN of the femoral head	1	2	
Heterotopic ossification	1	0	
Superior gluteal artery injury	0	1	
Post-traumatic arthritis	3	0	

AVN, avascular necrosis; M±SD, mean ± standard deviation; n, patient number; n/a, not applicable.

^a^ Independent *t* test or chi-square test.

^b^ Fisher's exact test.

*∗*The same patient sustained superior gluteal artery injury and AVN of the femoral head.

## Data Availability

The data that support the findings of this study are available on request from the corresponding author, Tsu-Te Yeh. The data are not publicly available because they contain information that could compromise the privacy of research participants.
